# MANAGEMENT OF NEURODEGENERATIVE DISEASES: APPLICATION OF MULTIMODAL, EXPERT-INTERACTIVE AI VERTICAL MODELS

**DOI:** 10.1093/geroni/igae098.0352

**Published:** 2024-12-31

**Authors:** Junyan Mao, Jialiang Tong, Weidong Huang, Hongyu Li

**Affiliations:** Shanghai Izhaohu Medical Technology Co. Ltd, Shanghai, Shanghai, China (People’s Republic); Shanghai Izhaohu Medical Technology Co. Ltd, Shanghai, Shanghai, China (People’s Republic); Changchun University of Chinese Medicine, Changchun, Jilin, China (People’s Republic); Jinzhou Medical University, Jin Zhou, Liaoning, China (People’s Republic)

## Abstract

This study proposes a comprehensive management model for neurodegenerative diseases, leveraging multimodal, expert-interactive AI vertical models to tackle the challenges of environmental dependency, temporal constraints, and data singularity. The model aims to enhance patient prognosis, quality of life, and survival. Our research methodology unfolds in three phases: The first step involves conducting a bibliometric analysis to integrate findings from existing studies. The second phase “Early Detection and Prediction,” leverages computer vision (CV) and natural language processing to gather multimodal biomarker data, including voice, gait, vision, hearing, and PET scans. Data is analyzed using machine learning (such as SustaIn, Random Forests, and Support Vector Machines) and deep learning techniques for robust data modeling. The final step, “Effectiveness Verification,” entails executing randomized controlled trials (RCTs) with a triple-blind setup—ensuring blindness among participants, researchers, and assessors—to rigorously evaluate the developed model’s effectiveness. The research involved five-year data from 304 individuals aged 65 and older with neurodegenerative diseases. Objective biomarker data were collected through CV and sensors to facilitate early identification and diagnosis of neurodegenerative diseases. A sophisticated platform facilitated real-time monitoring of mental and behavioral symptoms and activities of daily living at disease onset, integrating both pharmacological and non-pharmacological interventions via advanced language models for holistic disease management. Utilizing dynamic video analysis and machine learning evaluations, the model significantly mitigated the progression of cognitive and physical impairments, enhanced quality of life, and decreased mortality rates, affirming the preliminary efficacy of the comprehensive management model.

